# miRNA-128 modulates bone neoplasms cells proliferation and migration through the WNT/β-catenin and EMT signal pathways

**DOI:** 10.1186/s13018-020-02164-w

**Published:** 2021-01-20

**Authors:** Yang Li, Xiaotao Long, Ji Wang, Jing Peng, Kai Shen

**Affiliations:** Department of Orthopedics, Chongqing General Hospital, University of the Chinese Academy of Sciences, No. 312 Zhongshanyi Road, Yuzhong District, Chongqing, 400013 China

**Keywords:** miRNA-128, Bone neoplasms, Proliferation, Migration, Wnt/β-catenin, EMT

## Abstract

**Background:**

Bone neoplasms present poor prognosis due to recurrence and metastasis. Although the role microRNAs (miRNAs) in inhibiting growth and metastasis of bone neoplasms has been investigated, the underlying potential molecular mechanisms mediated by miRNA-128 (miR-218) for the invasiveness of bone neoplasms cells are still not completely understood. The purpose of this study was to identify the regulatory mechanisms of miR-218 in bone neoplasms cells.

**Methods:**

Western blotting, quantitative reverse transcription-polymerase chain reaction (qRT-PCR), Counting Kit-8 assay, terminal deoxynucleotidyl transferase-mediated dUTP nick end labeling (TUNEL) staining, luciferase activity assay immunofluorescence, and immunohistochemistry were used to analyze the regulatory effects of miR-218 on bone neoplasms cells.

**Results:**

Here, the results showed that transfection of miR-128 suppressed bone neoplasms cells proliferation, migration, and invasion. Genetic knockdown of miR-128 in bone neoplasms cells suppressed the activation of the Wnt/β-catenin and epithelial-mesenchymal transition (EMT) signaling pathways. Activation of Wnt or EMT blocked miR-128-inhibited cells proliferation and migration in bone neoplasms cells. Exogenously introduced miR-128 markedly inhibited tumor regeneration in bone neoplasms xenograft models.

**Conclusions:**

These results define a tumor-regulated function for miR-128 in bone neoplasms by down-regulation of the Wnt/β-catenin and EMT signal pathways, which provided a potential target for bone neoplasms gene therapy.

## Introduction

Bone neoplasms originate from bone and rapidly spread to the rest of tissues in the patients [1]. The main reason for the poor prognosis of bone neoplasms is recurrence and metastasis, and the 5-year survival rate of bone neoplasms is less than 30% [2]. At present, the issue of controlling the early metastasis of bone neoplasms has become a bottleneck in the treatment of this disease [3, 4]. Although many therapeutic protocols such as surgical therapy, chemoradiotherapy, and immunotherapy have been applied in bone neoplasms patients, the prognosis of clinical patients remains poor for short of effective therapeutic targets [5–9].

MicroRNAs (miRNAs) are endogenous, small noncoding RNAs with 18–25 nucleotides, which can transcribe from nonprotein coding genes or introns, and play multifunctional roles by regulating gene expression at a posttranscriptional level through binding to the 3′-untranslated regions of target genes [10–12]. Many reports have shown that proliferation and metastasis of human tumor cells are accompanied by abnormal expression of miRNAs [13–15]. Recently, miRNAs have been reported to be involved in tumor cells growth, proliferation, migration, differentiation, apoptosis aging, and death [16–19]. Ectopic expression of multiple miRNAs has been found during bone metastasis by targeting important osteoclast genes [20]. Results indicate that miRNA-128 (miR-128) is more highly expressed in drug-resistant breast cancer samples compared to drug-sensitive samples, and the decrease of miRNA-128 enhances the sensibility of breast cancer cells to chemodrugs by targeting B cell lymphoma 2-associated X (Bax) [21]. Findings also have showed that miR-128 is reduced in prostate cancer, and exogenously introduced miR-128 suppresses tumor regeneration in multiple prostate cancer xenograft models by targeting the stem cell regulatory factors B cell-specific Moloney murine leukemia virus insertion site 1 (BMI-1), NANOG, and transforming growth factor beta receptor 1 (TGFBR1) [22]. However, the mechanism by which miR-128 influences bone tumor cells osteoclast differentiation remains unclear.

Inactivation of the Wnt/β-catenin and epithelial-mesenchymal transition (EMT) signal pathways is involved in human osteosarcoma growth by arresting cell cycle in G2/M phase [23]. In addition, EMT signaling pathway plays an important role in osteosarcoma growth proliferation, and invasion [24–26]. In this study, we investigated the role of miR-128 in bone neoplasms cells proliferation and migration, and further explored the associations between miR-128 and tumor invasion-related signal pathways in bone tumor cells.

## Materials and methods

### Tissue samples

Bone cancer tissues and matched adjacent normal tissues were collected from 18 patients (9/9: male/female, age 57.5 ± 6.5 years old) after surgical resection. The expression of miR-128 of tissues was evaluated using quantitative reverse transcription-polymerase chain reaction (qRT-PCR).

### Cell line and cell culture

Bone cancer cell line Mg63 cells were purchased from BeNa Culture Collection (Shanghai, China) and cells were cultured in (Dulbecco’s modified Eagle medium (DMEM, Thermo Fisher Scientific, Inc.) supplemented with 10% fetal bovine serum (FBS, Thermo Fisher Scientific, Inc.). Normal bone cell line hFOB1.19 was a gift from Tumor Pathology Laboratory, Chongqing Medical University, and cultured in DMEM supplemented with 10% FBS, 1% penicillin, and 1% streptomycin. All cells were cultured at 37 °C in a humidified incubator with 5% CO_2_.

### qRT-PCR

Expression of miR-128 was evaluated using qRT-PCR. Briefly, total RNA was extracted from tissues or cells using RNeasy kits (QIAGEN, Valencia, CA, USA). RNA was reversely transcribed into cDNA using miRNA-specific RT primers (Thermo Fisher Scientific Inc., Waltham, MA, USA). The used primer sequences (Invitrogen, Shanghai, China) were as follows: miR-128, 5′-TCCGATCACAGTGAACCGGT-3′ (forward) and 5′-GTGCAGGGTCCGAGGT-3′ (reverse); U6, 5′-CTCGCTTCGGCAGCACA-3′ (forward) and 5′-AACGCTTCACGAATTTGCGT-3′ (reverse). qRT-PCR was performed by a TaqMan miRNA qRT-PCR assays (Applied Biosystem, Waltham, MA, USA) with ABI-Prism 7300 System (Applied Biosystem, Waltham, MA, USA). Expression of miR-1258 was measured using 2^−ΔΔCt^ as described previously [27].

### Knockdown of miR-128

Si-RNAs sequences for miR-128 (si-miR-128; 5′-UCACAGUGAACCGGUCUCUUU-3′) and miR-mimic negative control (si-NC; 5′-UGGUUUACAUGUUUUCCUA-3′) were obtained from Invitrogen Co., Ltd. (Shanghai, China). Cells were transfected with si-miR-128 (50 nM) of or si-NC (50 nM) using Lipofectamine 3000 (Thermo Fisher Scientific, Inc., Waltham, MA, USA) according to the manufacturer’s instructions. After 96 h of transfection, cells were used for further analyses.

### Gene overexpression

DNA sequences of *Wnt and E-cadherin* were amplified using human bone cells cDNA library, and cloned into a plasmid expression vector pcDNA3.1 (Invitrogen, Carlsbad, CA, USA) to generate *Wnt* overexpressing (pWnt) or *E-cadherin* overexpressing plasmids (pE-cad). The miR-128 knockdown Mg63 cells were transfected with pWnt, pE-cad, or a control empty plasmid (Control) using a Lipofectamine 3000 (Thermo Fisher Scientific, Inc., Waltham, MA, USA). Cells were used for further analyses after 72 h of transfection.

### Cell proliferation assay

Cell proliferation was analyzed using Counting Kit-8 (CCK-8, Dojindo Molecular Technologies, Inc., Kumamoto, Japan) assays following the manufacturer’s instructions. In brief, Mg63 cells (1 × 10^3^) were seeded in 96-well plates and cultured at 37 °C for 72 h. After cultures, CCK-8 solution was added for 1 h at 37 °C. The optical density (OD) was recorded at 450 nm using a microplate reader (Multiscan FC; Thermo Fisher Scientific, Inc., Waltham, MA, USA). Cell proliferation was analyzed using Image-Pro 5.0 software (Media Cybernetics, Bethesda, MD, USA).

### TUNEL staining

Mg63 cells (1 × 10^4^) were fixed with paraformaldehyde (4%) at 25 °C for 30 min, and permeabilized with 0.1% Triton-X-100 at 25 °C for 5 min. Apoptosis of Mg63 cells was analyzed using terminal deoxynucleotidyl transferase-mediated dUTP nick end labeling (TUNEL) assay (Sigma, Germany) according to the manufacturer’s protocols. Briefly, cells were incubated with TUNEL for 1 h at 4 °C, washed with phosphate buffer saline (PBS), and then incubated with 5% 4′,6′-diamidino-2-phenylindole (DAPI, Sigma-Aldrich, St. Louis, MO, USA) for 15 min at 25 °C. Finally, images were captured using Olympus IX73 microscope (Tokyo, Japan), and the apoptosis rate of cells was calculated by the software of Developer XD 1.2 (Definiens AG, Munich, Germany).

### Cell cycle analysis

Mg63 cells (1 × 10^7^) were collected, washed with PBS, and fixed in ethanol (70%) for 2 h at 4 °C. Cells were then incubated with RNase A (10 mg/L) for 30 min at 37 °C, washed with PBS and subsequently incubated in PI (10 g/mL) for 1 h at 4 °C. Cell cycle distribution was analyzed using Flow cytometry. The results were quantified using ModFit software version 3.0 (BD Biosciences, San Jose, CA, USA).

### Cell migration and invasion assay

Mg63 cells (1 × 10^5^) were cultured in Matrigel-uncoated and -coated transwell inserts (8 μm pore size; Millipore). For migration assay, Mg63 cells at density of 1 × 10^5^/mL were placed into the upper chamber with the non-coated membrane. For invasion assay, Mg63 cells were suspended as a density of 1 × 10^5^/mL in 500 μL DMEM containing 10% FBS. The cells were then subjected to the tops of BD BioCoat Matrigel Invasion Chambers (BD Biosciences, San Jose, CA, USA) according to the manufacturer’s protocol. After 48 h of incubation, Mg63 cells were fixed in 4% paraformaldehyde at 25 °C for 15 min, and stained with 0.1% crystal violet dye (Sigma-Aldrich) at 25 °C for 15 min. The cells were counted at three randomly selected views using a light microscope (Olympus, Tokyo, Japan).

### Western blotting

The total cellular proteins were extracted from cultured Mg63 cells (1×10^7^) using radioimmunoprecipitation assay (RIPA) lysis buffer (Sigma-Aldrich, St. Louis, MO, USA). Cells were centrifuged at 12,000×*g* at 4 °C for 15 min, and supernatant was collected. Protein concentration was determined by the BCA Protein Assay kit (Thermo Fisher Scientific, Inc., Waltham, MA, USA). A total of 30 μg of protein was separated on 12.5% sulfate-polyacrylamide gel electrophoresis (SDS-PAGE), and bolted onto a polyvinylidene fluoride (PVDF) membrane (Thermo Fisher Scientific, Inc., Waltham, MA, USA). Protein was blocked using 5% bovine serum albumin (BSA, Sigma-Aldrich, St. Louis, MO, USA) at 25 °C for 2 h, and then incubated with primary antibodies including Wnt (ab228526), β-catenin (ab32572), E-cadherin (ab76319), vimentin (ab92547), fibronectin (ab45688), caspase-3 (ab13847), caspase-9 (ab2020068), bone morphogenetic protein-2 (BMP-2, ab14933), vascular endothelial growth factor (VEGF, ab53465), Cyclin D1 (ab16663), Cyclin E2 (, ab32103), and β-actin (ab8226) at 4 °C for 12 h. All primary antibodies were purchased from Abcam, Cambridge, MA, USA, and used at a dilution of 1:1000. Membranes were then incubated with HRP-conjugated goat anti-rabbit IgG mAb secondary antibodies (1:5,000, cat. no. 4410, Cell Signaling Technology. Inc., Danvers, MA, USA) at 25 °C for 2 h. The immunoreactivity was evaluated using ECL Western blotting kit (Beyotime Institute of Biotechnology, Beijing, China). The density of protein was analyzed using ImageJ software 2.0 (Bio-Rad, USA) and normalized to β-actin.

### Luciferase activity assay

To validate direct targeting of miR-128, *Wnt* 3′UTR containing predicted miR-128 binding sites was amplified, subcloned into pGL3-control vectors (Promega, Fitchburg, WI, USA), and co-transfected with mimic miRNAs in Mg63 cells. Wild-type (WT) Wnt reporter and mutant type (MUT) Wnt reporter with mutant miR-128 binding sites were constructed using QuikChange II Site-Directed Mutagenesis Kit (Agilent Technologies, Santa Clara, CA, USA) according to the manufacturer’s instructions. After 72-h transfection, relative luciferase activity was analyzed using dualluciferase reporter assay (Promega, Fitchburg, WI, USA) according to the manufacturer’s instructions.

### Tumor xenograft assay

A total of 16 female nude mice at age of 6 weeks weighting 28–32 g were purchased from Animal Experimental Center of Chongqing Medical University (Shanghai, China). Mice were housed 23 ±0.5 °C, humidity of 50 ± 5%, a 12-h light/dark cycle, and free accessed to food and water in specific pathogen-free condition. Mg63 cells (1 × 10^5^) transfected with Si-miR-128 or Si-NC were subcutaneously injected into the right flank of mice (*n* = 8 per group). Mice were sacrificed using cervical decapitation on week 10, and tumors were isolated from xenograft, weighted, and used for immunohistochemistry (IHC) analysis.

### IHC analysis

Tissue samples were embedded in paraffin, cut into 4 μm thickness sections, deparaffinized in xylene, rehydrated through graded ethanols, and blocked in 3% hydrogen peroxide for 10 min at 25 °C as previously described [28]. Tumor sections were incubated with specific primary antibodies Wnt (1:1,000 dilution, ab228526), β-catenin (1:1,000 dilution, ab32572), E-cadherin (1:1,000 dilution, ab76319), vimentin (1:1,000 dilution, ab92547), fibronectin (1:1,000 dilution, ab45688) for 12 h at 4 °C. Tumor tissues were then incubated with secondary antibody (Alexa Fluor 488, 1: 2,000 dilution) at 25 °C for 2 h. All antibodies were purchased from Abcam, Cambridge, MA, USA. The results were captured at × 40 magnifications using the Olympus IX73 microscope (Tokyo, Japan).

### Statistical analysis

All data were presented as the mean ± standard deviation (SD). Statistical analyses were conducted using one-way analysis of variance (ANOVA) followed by Bonferroni’s adjustment where there were multiple comparisons in Statistical Product and Service Solutions software (SPSS 17.0 for Windows, SPSS, Chicago, IL, USA). Significance was accepted at *P* < 0.05.

## Results

### miR-128 expression was increased in bone neoplasms tissues and Mg63 cells

**Fig. 1:**
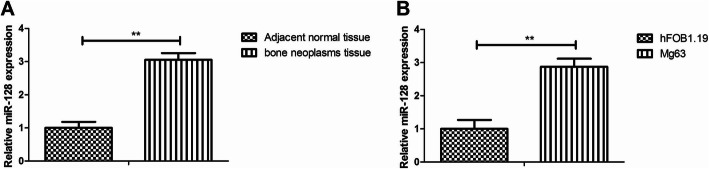
Expression of miR-128 in bone cancer tissues and Mg63 cells. **a** miR-128 expression in bone cancer tissues and adjacent normal tissues was measured. **b** miR-128 expression was measured by qRT-PCR in bone Mg63 cells and normal bone hFOB1.19 cells. **P < 0.01

To investigate the effect of miR-128 on bone tumor, its expression was accessed in bone neoplasms tissues and cell line Mg63 cells by qRT-PCR. The results revealed that miR-128 expression was higher in bone neoplasms tissues than adjacent normal tissues (*P* < 0.01; Fig. 1a). It was also demonstrated that miR-128 was highly expressed in bone neoplasm Mg63 cells compared to normal bone hFOB1.19 cells (*P* < 01; Fig. 1b).

### Knockdown of miR-128 inhibited proliferation, migration and invasion of Mg63 cells

**Fig. 2:**
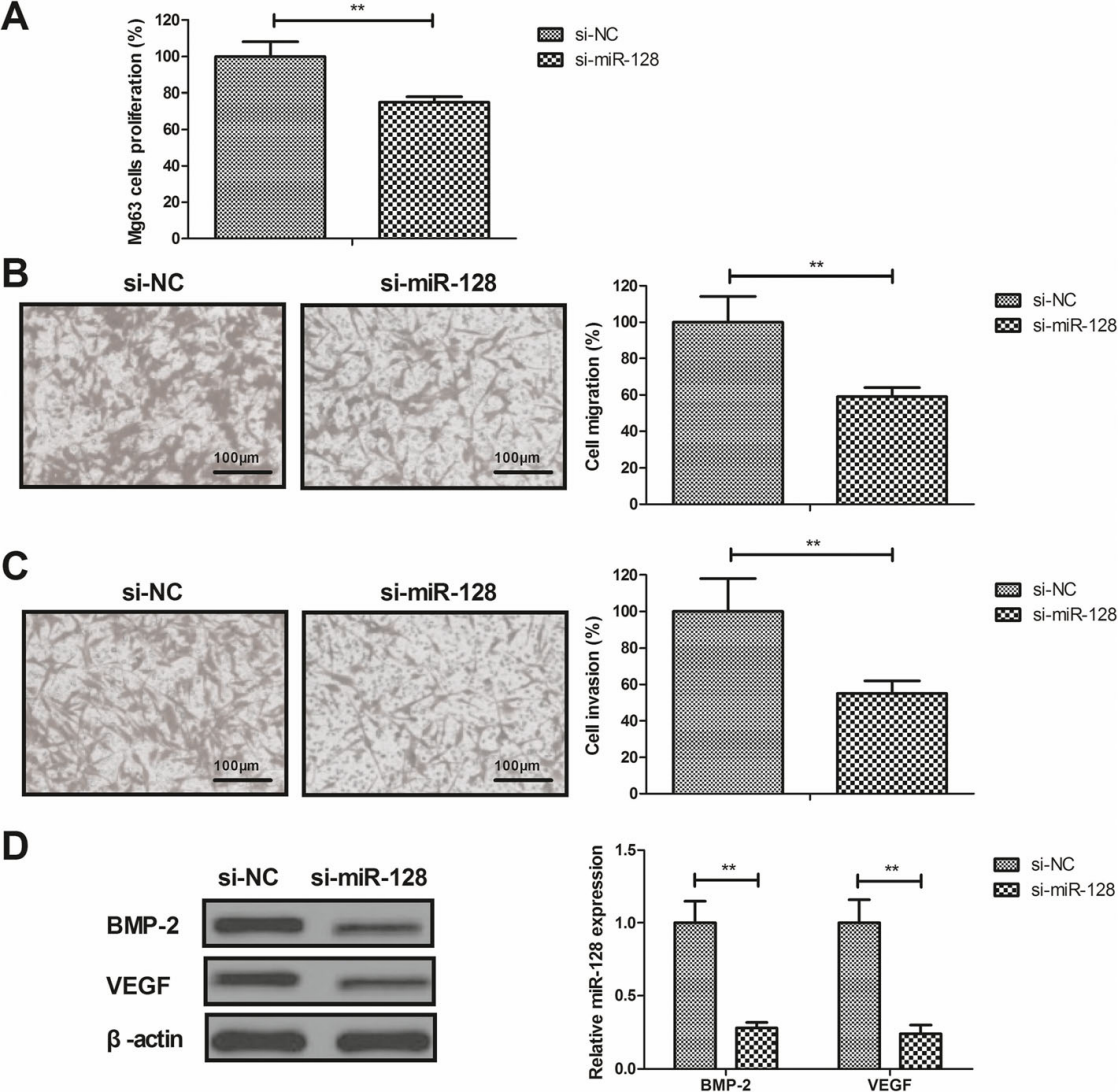
Knockdown of miR-128 inhibited Mg63 cells proliferation, migration, and invasion. Inhibitory effects of si-miR-128 transfection on proliferation (**a**), migration (**b**), and invasion (**c**) of Mg63 cells. **d** Protein expression of BMP-2 and VEGF in si**-**miR-128-transfected Mg63 cells. **P < 0.01 vs. si-NC

The anti-cancer effects of miR-128 were investigated in Mg63 cells in vitro. The results demonstrated that, compared to the control, knockdown of miR-128 inhibited Mg63 cells proliferation (*P* < 0.01; Fig. 2a), migration (*P* < 0.01; Fig. 2b), and invasion (*P* < 0.01; Fig. 2c). Furthermore, knockdown of miR-128 decreased BMP-2 and VEGF protein expression in Mg63 cells (*P* < 0.01; Fig. 2d).

### Knockdown of miR-128 induced apoptosis and arrested cell cycle of Mg63 cells

**Fig. 3:**
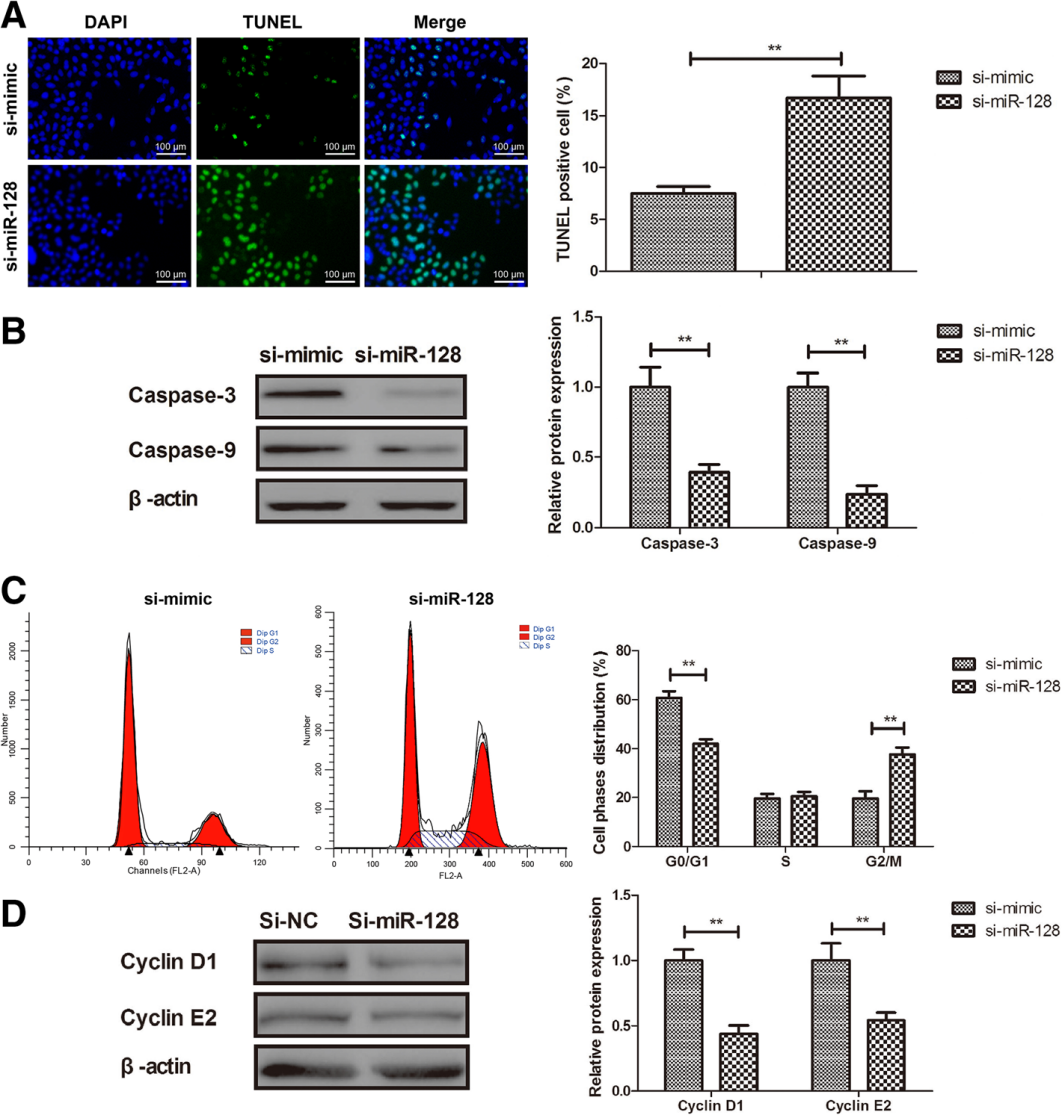
Knockdown of miR-128 induced apoptosis and arrested cell cycle of Mg63 cells. **a** si-miR-128 induced apoptosis of Mg63 cells. **b** si-miR-128 increased caspase-3 and caspase-9 expression in Mg63 cells. **c** si-miR-128 arrested G2/M phase in Mg63 cells determined by flow cytometry analysis. **d** Expression of cyclin D1 and cyclin E2 in si-miR-128-transfected Mg63 cells. **P < 0.01 vs. Si-NC

In order to examine the inhibitory effects of miR-128, apoptosis and cell cycle were evaluated in miR-128 knockdown Mg63 cells. As shown in Fig. 3, compared to the control, knockdown of miR-128 markedly induced apoptosis of Mg63 cells (*P* < 0.01; Fig. 3b) while it increased caspase-3 and caspase-9 protein expression in Mg63 cells (*P* < 0.01; Fig. 3b). Flow cytometry analyses demonstrated that miR-128 knockdown arrested G2/M phase in Mg63 cells (*P* < 0.01; Fig. 3c). Protein expression of Cyclin D1 and Cyclin E2 was downregulated in miR-128 knockdown Mg63 cells compared to miR-128 mimic-transfected Mg63 cells (*P* < 0.01; Fig. 3d).

### Knockdown of miR-128 suppressed the activation of the Wnt/β-catenin and EMT signaling pathways in Mg63 cells

**Fig. 4:**
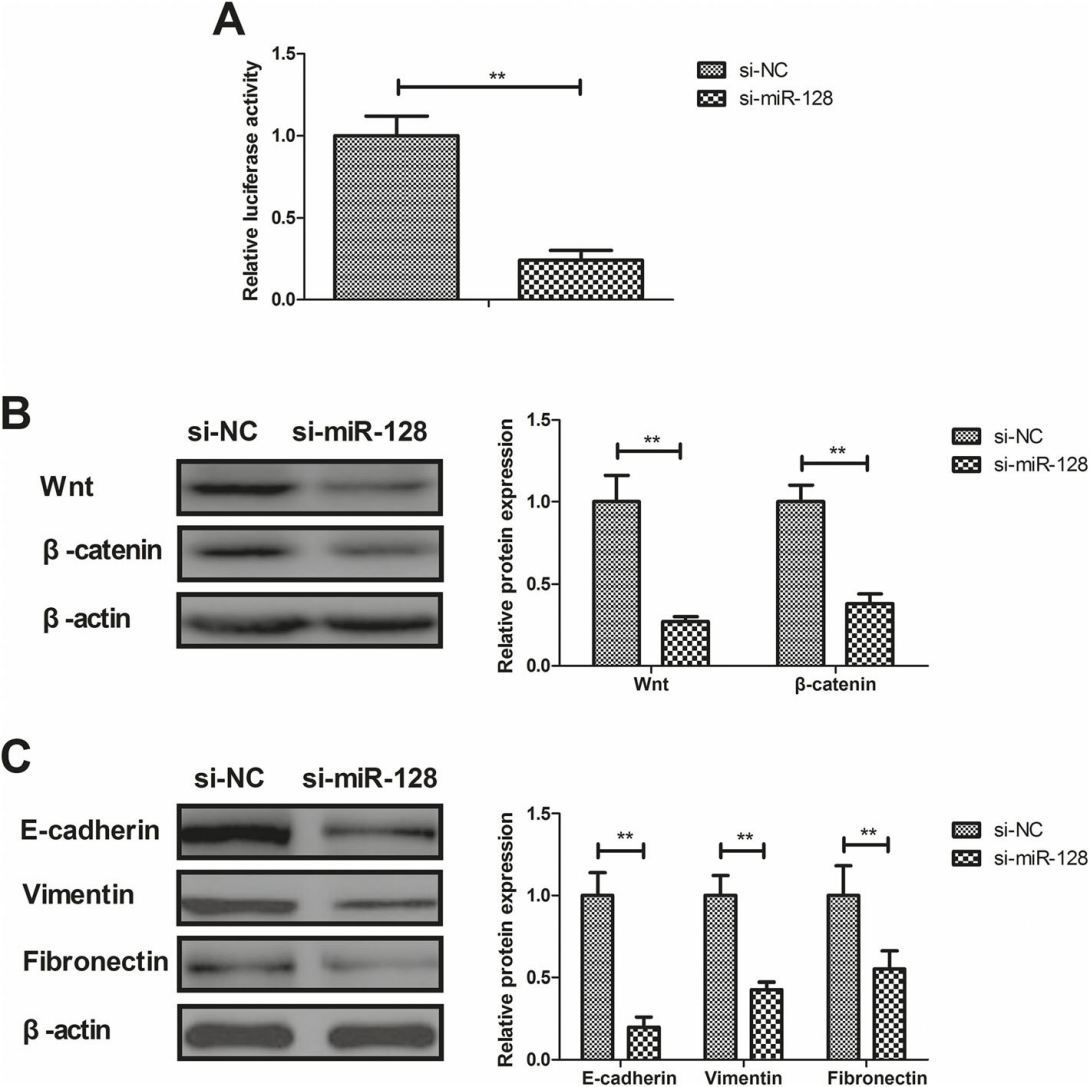
Si-miR-128 suppressed protein expression of Wnt/β-catenin and EMT in Mg63 cells. **a** Putative binding sites between Wnt 3′UTR and miR-128. **b** si-miR-128 decreased Wnt and β-catenin expression in Mg63 cells. **c** si-miR-128 decreased the epithelial markers E-cadherin and the mesenchymal markers vimentin and fibronectin in Mg63 cells. **P < 0.01 vs. si-NC

This study demonstrated whether knockdown of miR-128 changed the Wnt/β-catenin and EMT signaling pathways. Luciferase activity assay presented that relative luciferase activity was decreased in si-miR-128 Mg63 cells compared with si-NC Mg63 cells (*P* < 0.01), and miR-128 targeted to 3′UTR of Wnt (Fig. 4a). Immunoblotting analysis demonstrated that, compared to the control, knockdown of miR-128 downregulated Wnt and β-catenin protein expression (*P* < 0.01; Fig. 4b). Results also showed that genetic knockdown of miR-128 dramatically decreased the protein expression of the epithelial markers E-cadherin, and the mesenchymal markers vimentin and fibronectin in Mg63 cells (*P* < 0.01; Fig. 4c).

### Activation of Wnt/EMT blocked miR-128-inhibited cells proliferation and migration of Mg63 cells

**Fig. 5:**
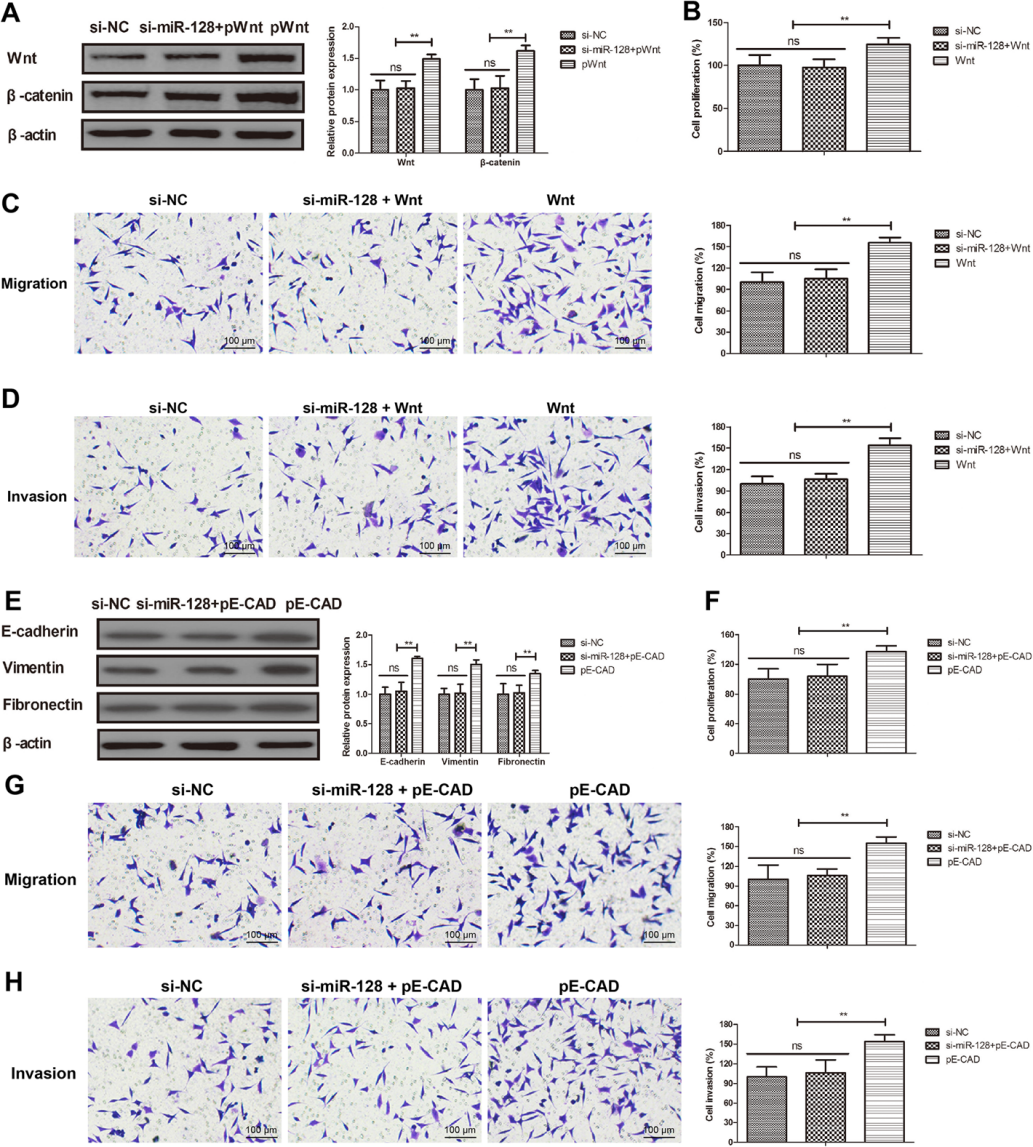
Activation of Wnt or EMT blocked miR-128-inhibited cells proliferation and migration of Mg63 cells. **a** Effects of pWnt on si-miR-128-decreased Wnt and β-catenin expression in Mg63 cells. Effects of pWnt on si-miR-128-inhibited proliferation (**b**), migration (**c**), and invasion (**d**) in Mg63 cells. **e** Effects of pE-cad on si-miR-128-decreased the E-cadherin, vimentin and fibronectin in Mg63 cells. Effects of pE-cad on miR-128-suppressed proliferation (**f**), migration (**g**), and invasion (**h**) in Mg63 cells. **P < 0.01

We further analyzed the role Wnt/EMT activation in miR-128-inhibited cell proliferation and migration in Mg63 cells. As indicated in Fig. 5a, Wnt and β-catenin expression was obviously promoted in Wnt overexpressed Mg63 cells. However, knockdown of miR-128 inhibited Wnt and β-catenin protein expression in Wnt overexpressed (pWnt) Mg63 cells (*P* < 0.01). Overexpression of Wnt blocked miR-128-inhibited proliferation (Fig. 5b), migration (Fig. 5c), and invasion (Fig. 5d) in Mg63 cells (all *P* < 0.01). Similarly, high expression of E-cadherin was found in E-cadherin overexpressed (pE-cad) Mg63 cells. si-miR-128 decreased protein expression of the epithelial markers E-cadherin and the mesenchymal markers vimentin and fibronectin in Mg63 cells (*P* < 0.01; Fig. 5e). Moreover, EMT activation canceled miR-128-suppressed proliferation (Fig. 5f), migration (Fig. 5g), and invasion (Fig. 5h) in Mg63 cells (all *P* < 0.01).

### Knockdown of miR-128 suppressed tumor growth in xenograft models

**Fig. 6:**
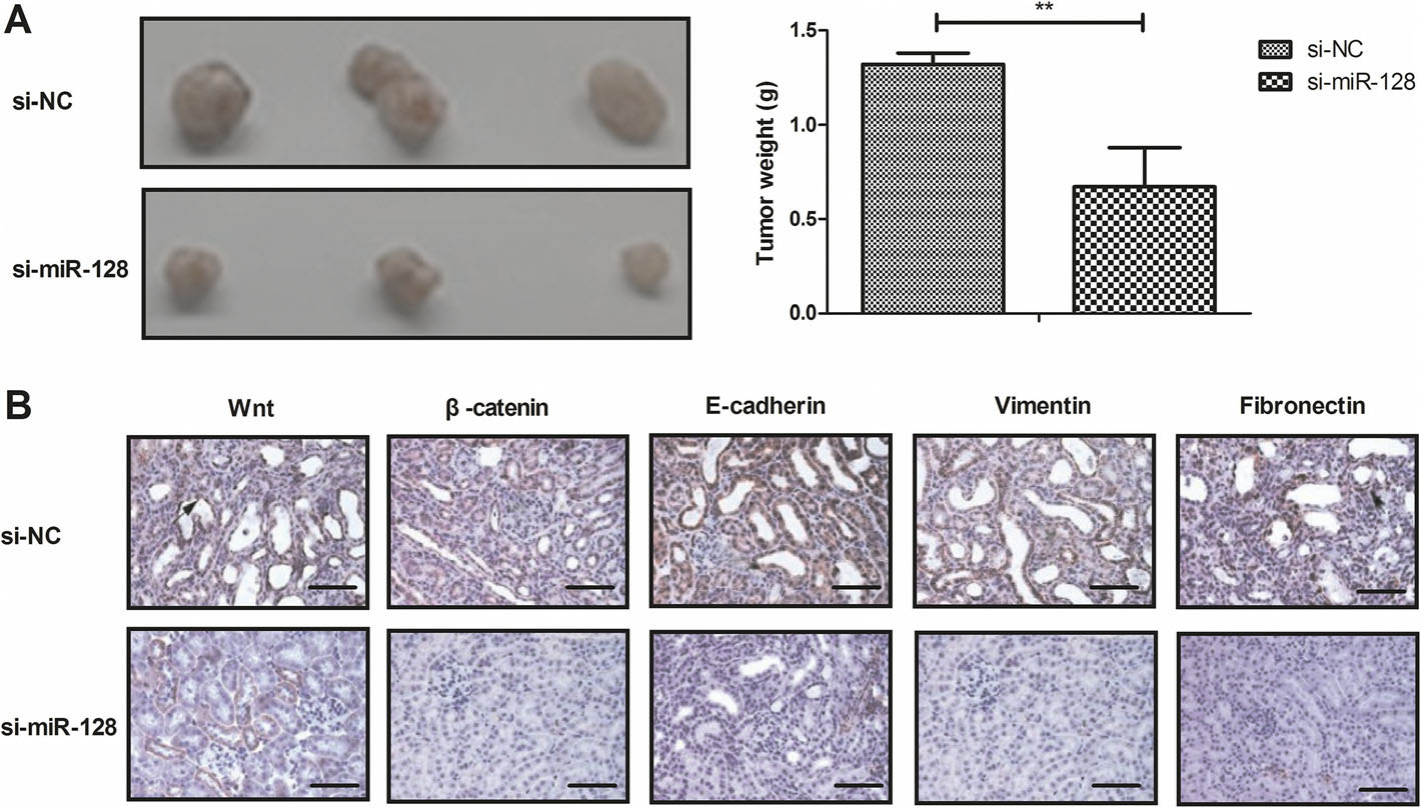
Si-miR-128 suppressed tumor growth in xenograft models. **a** Knockdown of miR-128 inhibited tumor growth in xenograft models during 10 weeks observation. **b** Knockdown of miR-128 decreased the Wnt/β-catenin and EMT signal pathways in tumor tissue. Magnification, × 40. **P < 0.01 vs. Si-NC

To determine whether miR-128 knockdown suppressed bone tumor growth, si-miR-128- or si-miR-mimic knockdown Mg63 cells were subcutaneously injected into the flanks of experimental mice. As shown in Fig. 6a, exogenous knockdown of miR-128 markedly inhibited tumor regeneration in bone neoplasms xenograft models during 10 weeks observation compared to the control mice (*P* < 0.01). Immunohistochemistry staining showed that miR-128 knockdown decreased expression of Wnt, β-catenin, E-cadherin, vimentin, and fibronectin, indicating that miR-128 knockdown inhibited the Wnt/β-catenin and EMT signaling pathways in tumor tissues of bone neoplasms (Fig. 6b).

## Discussion

miRNAs are involved in the diagnosis of OA as well as in its treatment [29, 30]. The data in a previous study indicated that the miR-128 downregulation inhibits osteosarcoma cell proliferation and migration phenotype [31]. Activation of Wnt/β-catenin signaling pathways play essential roles and may rescue chemotherapy drug resistance by targeting Beclin 1 in osteosarcoma cells [32]. Blocking EMT can suppress osteosarcoma cell proliferation by inducing apoptosis [33]. In this study, in vitro and in vivo data demonstrate that miR-128 expression is upregulated in both bone cancer tissue and Mg63 cells. Further, miR-128 has a regulatory function in bone cancer cell proliferation, metastasis, apoptosis, and tumor regeneration. Mechanistically, findings in the current study indicate that knockdown of miR-128 in Mg63 cells interferes with the Wnt/β-catenin and EMT signaling pathways. Of note, miR-128 knockdown inhibited cell growth, migration, and invasion partly through the suppression of the Wnt/β-catenin and EMT signaling pathways in Mg63 cells.

Currently, knockdown of miR-128 in breast tumor-initiating cells was reported to induce chemotherapeutic resistance, which contributes to chemotherapeutic resistance in breast cancers by targeting of Bmi-1 and ATP binding cassette subfamily C member 5 (ABCC5) [34]. Previous findings provided the evidence that miR-128 may serve as a potential therapeutic target in glioma by inhibition of tumor angiogenesis via p70S6K1 [35]. In addition, miR-128 enhances dendritic cell-mediated anti-tumor immunity via targeting p38 mitogen activated protein kinase (MAPK) signaling pathway in C57BL/6 mice bearing B16 melanoma [36]. Data in this study found that miR-128 knockdown inhibits bone cancer cells proliferation, migration, invasion, and promotes apoptosis and cell cycle. Results demonstrated that miR-128 knockdown effectively induced osteosarcoma cell apoptosis via decreasing caspase-3 and caspase-9 and arrested Mg63 cells at G2/M phase via inhibition of cyclin D1 and cyclin E2. The potential roles need to be further explored to clarify the regulatory function of miR-128 in bone tumor cells in future.

Studies have found that the Wnt/β-catenin and EMT signaling pathways are associated with tumor cell proliferation, apoptosis, and cell cycle regulation [37–39]. Aberrant activation of Wnt signaling has been implicated in human osteosarcoma, and Wnt/β-catenin signaling plays the critical role in human osteosarcoma pathogenesis and growth [37]. Inactivation of the Wnt/beta-catenin signaling inhibits angiogenesis and induces cell apoptosis in osteosarcoma cells by inducing autophagy [38]. Inhibition of EMT serves as a tumor suppressor in osteosarcoma by suppressing the progression and metastasis [39]. Furthermore, EMT inactivation inhibited proliferation, migration, invasion, and in osteosarcoma by targeting zinc finger E-box-binding protein 1 (ZEB1) through inactivation of c-Jun N-terminal kinase (JNK) and JAK1/signal transducer and activator of transcription 3 (STAT3) pathways [25]. Data in this study found that knockdown of miR-128 suppresses the activation of the Wnt/β-catenin and EMT signal pathways in bone neoplasms tissues and cells. However, activation of Wnt/EMT blocked miR-128-inhibited cells proliferation and migration of bone neoplasms cells. Notably, in vivo assay showed that disrupting miR-128 expression in Mg63 cells presents small tumor weight compared with miR-mimic transfected cells. The reduced growth of miR-128 knockdown tumors show suppression of Wnt/beta-catenin and EMT signaling pathways, suggesting inhibition of Wnt/beta-catenin and EMT might contribute to tumor growth inhibition. Therefore, further analyses are needed to clearly elucidate miR-128 knockdown mechanisms in vitro and in vivo.

In summary, data in the current study demonstrates that the high miR-128 expression is found in bone neoplasms tissues and cells. miR-128 knockdown inhibits bone cancer cell proliferation and metastasis, and tumor growth through suppression of the Wnt/β-catenin and EMT signaling pathways. It is indicated that targeting miR-128 may provide new avenues for the research of a potential therapeutic agent and clinical biomarker of bone cancer growth and metastasis.

## Data Availability

The analyzed data sets generated during the study are available from the corresponding author on reasonable request.

## Abbreviations

miR-218: miRNA-128
miRNAs: microRNAs
miR-128: miRNA-128
qRT-PCR: Quantitative reverse transcription-polymerase chain reaction
pWnt: Wnt overexpressing
pE-cad: E-cadherin overexpression plasmid
PVDF: Polyvinylidene fluoride
WT: Wild-type
MUT: Wnt reporter and mutant type
EMT: Epithelial-mesenchymal transition
TUNEL: Terminal deoxynucleotidyl transferase-mediated dUTP nick end labeling
Bax: B cell lymphoma 2-associated X
BMI-1: B cell-specific Moloney murine leukemia virus insertion site 1
TGFBR1: transforming growth factor beta receptor 1
SD: Standard deviation
ANOVA: One-way analysis of variance
SPSS: Statistical Product and Service Solutions software
IHC: Immunohistochemistry
RIPA: Radioimmunoprecipitation assay
DAPI: 4′,6′-diamidino-2-phenylindole
PBS: Phosphate buffer saline
SDS-PAGE: Sulfate-polyacrylamide gel electrophoresis
BSA: Bovine serum albumin
DMEM: Dulbecco’s modified Eagle medium
FBS: Fetal bovine serum
MAPK: Mitogen-activated protein kinase
ZEB1: Zinc finger E-box-binding protein 1
JNK: c-Jun N-terminal kinase
STAT3: Signal transducer and activator of transcription 3
